# ﻿*Commelinadanxiaensis* (Commelinaceae), a new species from Guangdong, China

**DOI:** 10.3897/phytokeys.218.91199

**Published:** 2023-01-13

**Authors:** Long-Yuan Wang, Wan-Yi Zhao, Zai-Xiong Chen, Wei-Cheng Huang, Ming-Yan Ding, Jin-Chu Luo, Wen-Bo Liao, Wei Guo, Qiang Fan

**Affiliations:** 1 Department of Horticulture and Landscape Architecture, Zhongkai University of Agriculture and Engineering, Guangzhou, Guangdong, China Zhongkai University of Agriculture and Engineering Guangzhou China; 2 State Key Laboratory of Biocontrol and Guangdong Provincial Key Laboratory of Plant Resources, School of Life Sciences, Sun Yat-sen University, Guangzhou, Guangdong, China Sun Yat-sen University Guangzhou China; 3 Administrative Commission of Danxiashan National Park, Shaoguan, Guangdong, China Administrative Commission of Danxiashan National Park Shaoguan China; 4 Shunde Polytechnic, Foshan, Guangdong, China Shunde Polytechnic Foshan China

**Keywords:** Commelinales, morphology, Mount Danxia, phylogeny, taxonomy

## Abstract

*Commelinadanxiaensis* (Commelinaceae), a remarkable new species from Mount Danxia, Guangdong Province, China, is described and illustrated. This species is similar to *C.communis* in inflorescences and flowers but readily distinguishable in its nearly erect stems, larger flowers, and different petal colouration.

## ﻿Introduction

*Commelina* L. is the largest genus of Commelinaceae, consisting of about 170 species distributed in tropical and subtropical regions (Faden 1998, 2012), with eight species recorded for China (Hong 1997; Hong and DeFilipps 2000). The genus is most diverse in tropical Africa, where an estimated 100 species occur (Faden 2001), with a wide range of variation in habit, inflorescence, flower colour, capsule dehiscence, seed number, testa ornamentation, and chromosome number (Faden 2012). Most of its species occur in open environments or as weeds along roadsides or in cultivation fields, but a few of them are restricted or exclusively known from forest habitats (Faden 2012; Nandikar 2013; Nandikar and Gurav 2014). *Commelina* is easily differentiated from the remaining genera in the tribe by its inflorescences which are subtended by spathaceous basal bracts and reduced to (1–)2 fasciculate cincinni, zygomorphic flowers, petals clawed, unequal and mostly blue (but sometimes white or lilac, rarely yellow, apricot or orange), three posterior staminodes with 6-lobed cruciform antherodes, three anterior stamens, and 2-locular or unequally 3-locular and 2-valved capsules (Faden 1998; Pellegrini and Forzza 2017).

During our botanical investigation of Mount Danxia of Guangdong Province in 2019–2020, we found an unusual population of *Commelina*. The plants are most closely similar to *C.communis* L., a species widely distributed in Asia, but differ mainly by the ascending stems (vs. creeping), petals sky-blue with a white basal third (vs. evenly dark blue).

## ﻿Materials and methods

Morphological observations of the putative new species and its close relatives were carried out based on living plants in the field, as well as on dried specimens. The voucher specimens were deposited in the Herbarium of Sun Yat-sen University (**SYS**). Leaf samples for the putative new species were collected and stored in silica gel. Total DNA was extracted from dried leaves using the modified CTAB method following the protocol of Doyle and Doyle (1990). In order to clarify the taxonomic delimitations of the putative new species and its relative species, sequences from one nuclear gene (nrITS) and three chloroplast DNA regions (*matK*, *rbcL* and *trnH*-*psbA*) were used respectively to estimate a Maximum Likelihood phylogeny tree. Some species belonging to genera of tribe *Commelineae*, which were related to *Commelina* (Faden, 1991), have been chosen as outgroups, such as *Floscopascandens* Lour., *Murdanniaedulis* (Stokes) Faden, and *M.bracteata* (C.B.Clarke) Kuntze ex J.K.Morton, sequences of which were downloaded from Genbank. Details of sequence data are given in Appendix 1.

The multiple sequences of each gene were aligned with Clustal X (Thompson et al. 1997) and then manually adjusted in Sequence Alignment Editor (Rambaut 2002). Gaps within the sequence were treated as missing data. The whole dataset was analysed with Maximum Likelihood (ML) methods. Clade support was estimated using 1,000 bootstrap replicates (BS) with the same heuristic search conditions. The ML analyses were performed with RAxML v.8.2.4 (Stamatakis 2014).

## ﻿Results

### ﻿Molecular analyses

**Figure 1.**
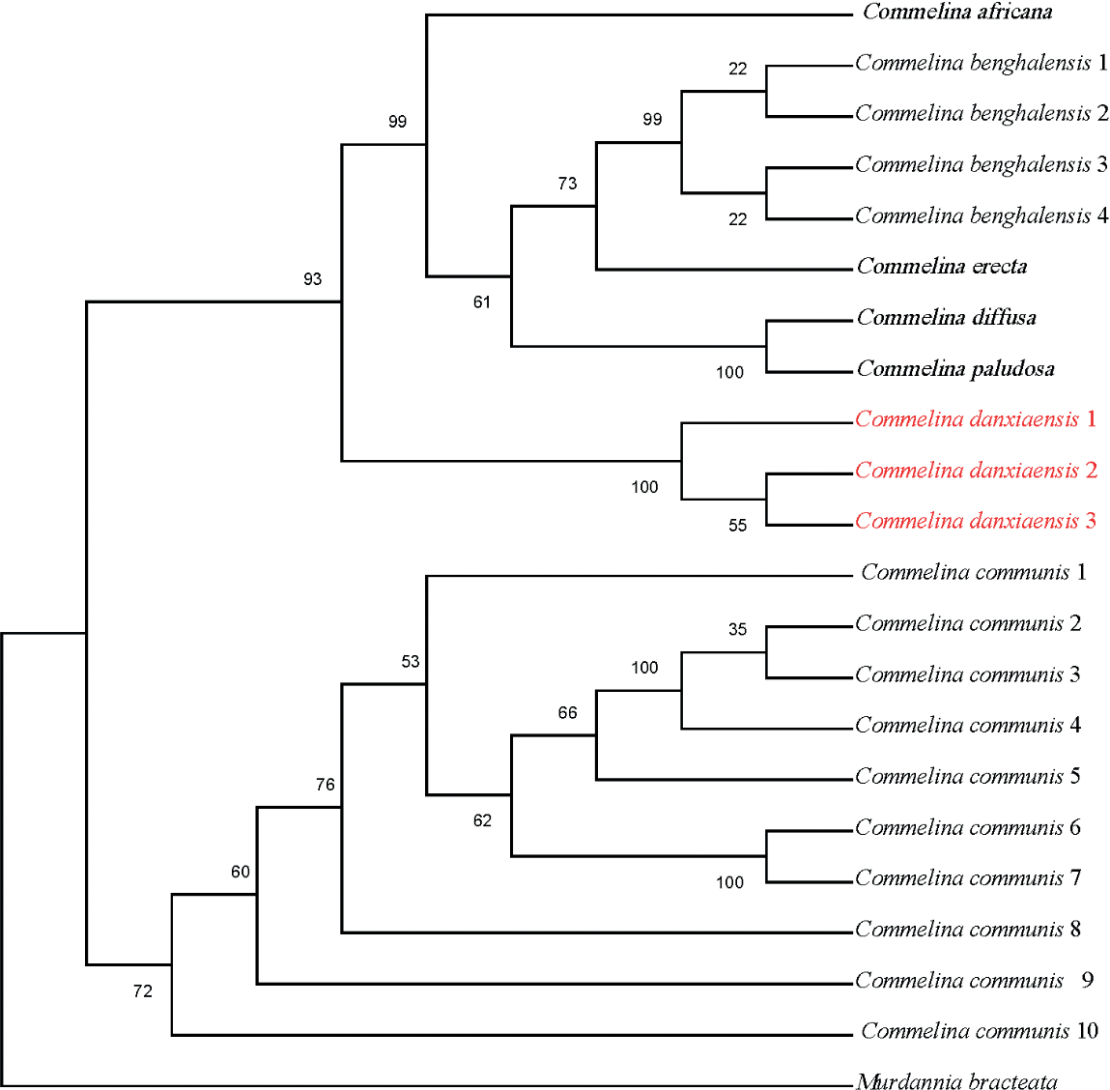
Maximum Likelihood consensus tree of the new species and related species based on nrITS sequence. Numbers beside nodes represent ML bootstrap values. The accessions for the new species are shown in red colour.

**Figure 2.**
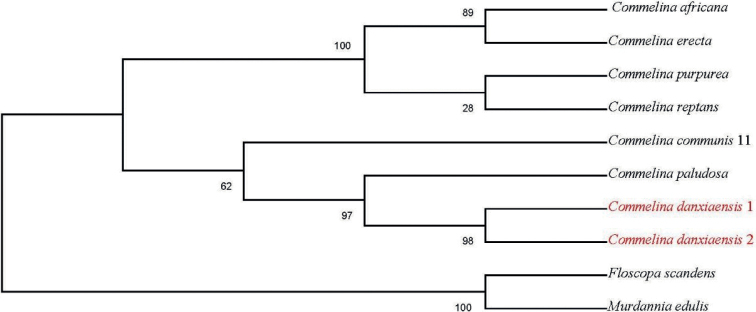
Maximum Likelihood consensus tree of the new species and related species based on three chloroplast sequences (*matK*, *rbcL*, and *trnH-psbA*). Numbers beside nodes represent ML bootstrap values. The accessions for the new species are shown in red colour.

The ML phylogenetic tree based on nrITS (Fig. 1) showed that all seven *Commelina* species were clustered into one lineage. Three individuals of *Commelinadanxiaensis* were recovered in a clade sister to a clade composed of *C.africana*, *C.benghalensis*, *C.erecta*, *C.diffusa*, and *C.paludosa*. Ten individuals of *C.communis* are recovered in a poorly supported clade, distantly related to *C.danxiaensis*. The ML phylogenetic tree based on three chloroplast DNA regions (*matK*, *rbcL*, and *trnH*-*psbA*) (Fig. 2) showed that all seven *Commelina* species were clustered into one lineage, and the outgroups were clustered into another one. And the two individuals of *C.danxiaensis* clustered into one lineage and were sistered to the lineage clustered with *C.communis* and *C.paludosa*. Although the phylogenetic trees based on nrITS and chloroplast DNA were in conformity, the individuals of *C.danxiaensis* formed one lineage, which partly supported it as a distinct species.

### ﻿Taxonomic treatment

#### ﻿ Commelina danxiaensis

Taxon classificationPlantaeCommelinalesCommelinaceae

Q.Fan, Long Y.Wang & W.Guo sp. nov.

1BDA1A62-7892-57D7-B50A-8B9C95A17CE7

urn:lsid:ipni.org:names:77311811-1

**Figure 3.**
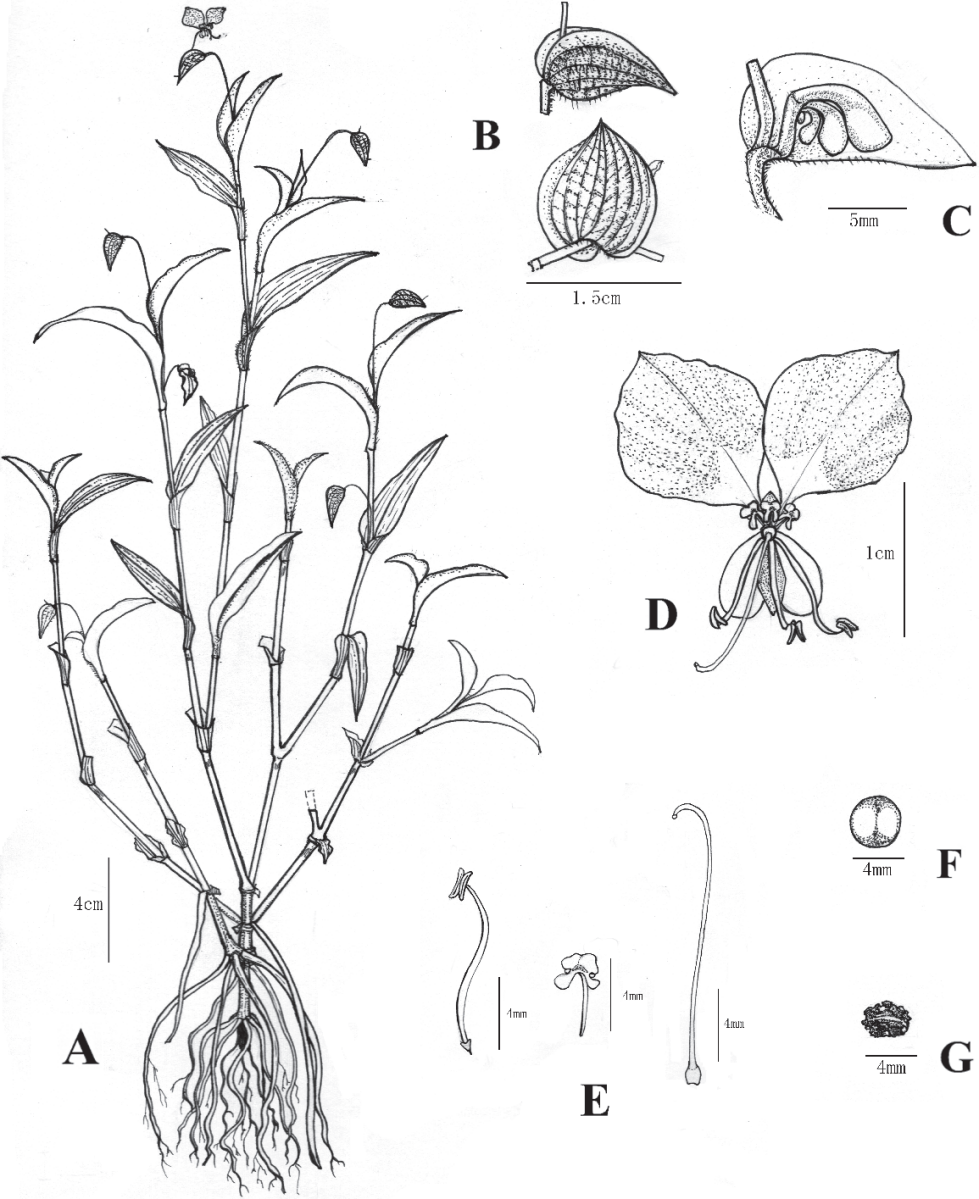
*Commelinadanxiaensis* sp. nov. **A** habitat **B** involucral bract **C** inflorescence **D** flower **E** fertile, sterile stamens and pistil **F** fruit in transverse section **G** seed.

**Figure 4.**
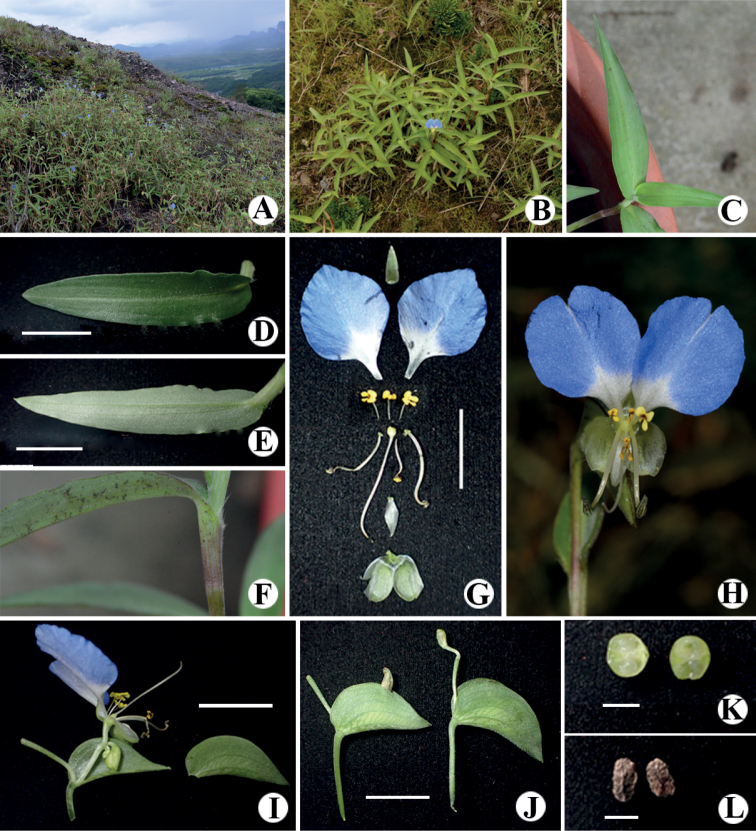
*Commelinadanxiaensis* sp. nov. **A** habitat, growing on top of the Danxia landform **B** habit, plants ascending **C–E** leaf-blade, lanceolate to ovate-lanceolate **F** leaf-sheaths, with pubescence **G–H** flower **I** inflorescence **J** spathe **K** fruit in transverse section, 2 seeds per valve **L** seed. Scale bars: 1 cm (**D, E, G, I, J**); 3 mm (**K, L**).

Figs 3 , 4 Chinese name. 丹霞鸭跖草 [dān xiá yā zhǐ cǎo]

##### Type.

China. Guangdong: Shaoguan City, Renhua County, Mount Danxia, 24°56'29.73"N, 113°45'21.96"E, dry mountaintop, ca. 350 m elev., 23 May 2020, *Q. Fan 17910* (holotype: SYS!; isotypes: SYS!, IBSC!).

##### Diagnosis.

*Commelinadanxiaensis* is morphologically similar to *C.communis* due to their diffuse stems, lanceolate leaf-blades and approximate floral forms. However, it differs in its ascending stems (Fig. 4B), tomentose leaf-blades and sheaths (Fig. 4C–F), and sky-blue lateral petals with a white basal third (Fig. 4G).

##### Description.

***Herbs*** over 30 cm tall, annual. ***Roots*** thin, fibrous. ***Stems*** ascending, many-branched; young shoots tomentose, becoming glabrous when old, hairs hyaline. ***Leaves*** spirally-alternate, evenly distributed along the stems; sheaths tomentose, hairs hyaline, margin ciliate, hairs setose, white; blades 3–9 × 1.5–2.0 cm, lanceolate to ovate-lanceolate, tomentose on both sides, evently, hairs hyaline, base obtuse, margin glabrous, green to light-purple,, apex lanceolate. ***Inflorescences*** leaf-opposite, restricted to the apex of the stems; peduncle 1.5–4.0 cm long, tomentose, hairs hyaline; spathe 1.2–2.5 × 1.0–2.2 cm, cordate, 6–8 veined, adaxially tomentose, long pilose hairs sometimes along the veins, hairs hyaline, abaxially glabrous, base cordate, free, margin green, apex acute; upper cincinnus developed, 1-flowered, flower staminate, peduncle ca. 8 mm long, exerted, sparsely tomentose to nearly glabrous, hairs hyaline; lateral cincinnus 2–3-flowered, flowers bisexual, peduncle 5–8 mm long, nearly included, tomentose to nearly glabrous, hairs hyaline. ***Flowers*** chasmogamous, bisexual or staminate, strongly zygomorphic; pedicels c. 3 mm long at anthesis, less than 6 mm long in fruit, reflected, nearly glabrous, hairs hyaline; sepals green, membranous, glabrous, dorsal sepal c. 5 × 2 mm, triangular-lanceolate, apex lanceolate, lateral sepals c. 5 mm × 3 mm, widely oblique-elliptic, connate for 3.6–4.2 mm, apex obtuse to round; paired petals short-clawed, claw c. 2–3 mm long, limb c. 1 cm × 1 cm, orbicular to sub-orbicular, sky-blue with a white basal third, medial petal c. 5 × 2 mm, narrowly rhombic, white, nearlly hyaline; staminodes 3, subequal, the medial slightly shorter, filaments c. 3 mm long, antherodes X-shaped, distinctly four-lobed, upper lobes c. 1/2 to the lower ones, yellow, with a dark maroon at centre; lateral stamen filaments ca. 1 cm long, curved, anthers 1.8–2.2 × 1.2–1.4 mm, elliptic; medial stamen filament ca. 5 mm long, anther 2.2–2.4 × 1.8–2.2 mm, saddle-shaped; ovary c. 1 × 1 mm, ovoid, glabrous, 2-locular, ovules 2 per locule, style c. 1.2 cm long, strongly curved at apex, stigma trilobate, white. ***Capsule*** c. 5 × 3 mm, ellipsoid, glabrous, equally 2-valved. ***Seeds*** 2 per valve, 3–4 × 2–2.5 mm, elliptic, ventrally flattened, testa brown, irregularly pitted.

##### Phenology.

*Commelinadanxiaensis* flowers from April to July and fruits from June to September.

##### Distribution and habitat.

*Commelinadanxiaensis* is only known from the type locality, Mount Danxia, Renhua County, Guangdong Province, China. Only two populations have been found, with several hundred individuals. It grows on dry mountaintops of the Danxia formation at elevations of 100–350 m.

##### Conservation assessment.

*Commelinadanxiaensis* has only been found in Mount Danxia within an area of less than 20 km^2^, making it putatively ‘Vulnerable’ (VU D2) according to the guidelines of the IUCN Red List Categories and Criteria (IUCN 2022). However, the threat risk seems low because it is not economically valuable, and the area’s conservation is good.

##### Additional specimens examined

**(*paratypes*).** China. Guangdong: Shaoguan City, Renhua County, Mount Danxia, fl., 3 July 2020, *Q. Fan 18026* (SYS); loc. cit., fr., 29 August 2020, *Q. Fan 18231* (SYS); loc. cit., fr., 29 August 2020, *Q. Fan 18232* (SYS).

##### Discussion.

The new species *Commelinadanxiaensis* is similar to *C.communis* and *C.diffusa* in some of its morphological characters, such as lanceolate leaf-blades, cordate spathes free at base, and blue paired petals. Morphological comparisons among them have been listed in Table 1.

**Table 1.** Morphological comparison amongst *Commelinadanxiaensis*, *C.communis*, *C.diffusa* and *C.bicolor* D.Q. Wang & M.E. Cheng.

Character	*C.danxiaensis*	*C.communis*	*C.diffusa*	*C.bicolor* D.Q. Wang & M.E. Cheng
Stem	ascending	creeping	creeping	unknown
Phyllotaxy	spirally-alternate	distichously-alternate	distichously-alternate	unknown
Leaf-blades	tomentose	glabrous	glabrous or hispid	unknown
Leaf-sheaths	tomentose	glabrous	hispid or hispid-ciliate	glabrous
Flower size	2.5 × 2 cm	2 × 1.5 cm	2 × 1.5 cm	unknown
Lateral sepals connation	connate	connate	free	unknown
Lateral petals colour	sky blue, basal third white	dark blue, sometimes lilac or white	light blue, sometimes lilac	upper most part deep-blue, basal part with claws (3 mm long) white
Medial petal colour	white	white	light blue	white
Medial petal claw	absent	absent	present	absent
Medial petal shape	narrowly rhombic	obtrullate	rhombic	oribicular-ovata or broadly ovata
Medial staminode development	developed	developed	not developed	developed
Stigma colour	white	white	white	unknown
Capsule valve number	equally 2-valved	equally 2-valved	unequally 2-valved	2-valved
Seed number per valve	2-seeded	2-seeded	dorsal valved 1-seeded, ventral valve 2-seeded	2-seeded
Seed testa ornamentation	irregularly pitted	irregularly pitted	Double-reticulate	irregularly foveolate

Additionally, we found the name *Commelinabicolor* D.Q.Wang & M.E.Cheng described by Wang et al. (2019), which is a later homonym of *C.bicolor* Poepp. ex Kunth (1843). Therefore, it is illegitimate under Art. 53.1 of ICN (Turland et al. 2018). This species is documented in Anhui and Hubei Provinces, and the type specimen (*D.Q. Wang, Y.Y. Lu & L. Zhang W1406291*, PE) was collected at Daqian Shan of Feixi County in Anhui Province (Wang et al. 2019). We previously believed the unknown species of *Commelina* in Mount Danxia to be conspecific with *C.bicolor* due to its similar morphological and ecological characteristics and treated it as a new distribution record for the Guangdong flora (Dai et al. 2021). However, we have been unable to check the type material at PE, where the authors stated the holotype specimen was deposited, to confirm the plant’s identity and the name’s application. We carried out fieldwork at Daqian Shan but were unable to find any individuals of this species. Hence, treatment has not been made for this species at the moment. It is planned that a further study will be carried out in the future.

Up to the present time, four species of *Commelina* have been documented in Danxiashan National Nature Reserve, including the new species *Commelinadanxiaensis*. In order to facilitate identification, we here provide a key and checklist for the *Commelina* species in Mount Danxia.

### ﻿Key to the *Commelina* species in Mount Danxia

**Table:** 

1	Spathe margin connate at base, base truncate	***C.benghalensis***
–	Spathe margin free at base, base cordate or rounded	**2**
2	Spathe narrowly cordate, veins concolourous to the spathe and inconspicuous; medial petal subequal to the laterals; capsules unequally 2-valved	***C.diffusa***
–	Spathe cordate, veins dark green and conspicuous; medial petal unequal to the laterals; capsules equally 2-valved	**3**
3	Stem ascending; lateral petals sky blue, basal third white	***C.danxiaensis***
–	Stem prostrate; lateral petals evenly dark blue, sometimes lilac or white	***C.communis***

## Supplementary Material

XML Treatment for Commelina danxiaensis

## ﻿Appendix 1

**Table A1.** Details of sequence data and taxa arranged for phylogeny research.

Species sequenced	GenBank accessions				Voucher information
	nrITS	matK	rbcL	trnH-psbA	
*Commelinaafricana*	KR733806.1	KR734981.1	KR737249.1	KR736019.1	/
*Commelinabenghalensis* 1	OQ189896	OQ164772	OQ164778	OQ164784	*L.Y. Wang 1833* (SYS), Guangzhou
*Commelinabenghalensis* 2	MH768093.1	/	/	/	/
*Commelinabenghalensis* 3	MH768094.1	/	/	/	/
*Commelinabenghalensis* 4	ON908416.1	/	/	/	/
*Commelinacommunis* 1	OM934863.1	/	/	/	/
*Commelinacommunis* 2	MZ489676.1	/	/	/	/
*Commelinacommunis* 3	OM934871.1	/	/	/	/
*Commelinacommunis* 4	MH703263.1	/	/	/	/
*Commelinacommunis* 5	OM934864.1	/	/	/	/
*Commelinacommunis* 6	OM934865.1	/	/	/	/
*Commelinacommunis* 7	OM934867.1	/	/	/	/
*Commelinacommunis* 8	OM934870.1	/	/	/	/
*Commelinacommunis* 9	MH710676.1	/	/	/	/
*Commelinacommunis* 10	OM934869.1	/	/	/	/
*Commelinacommunis* 11	/	OQ164773	OQ164779	OQ164785	*Q. Fan 18145* (SYS), Renhua
*Commelinadanxiaensis* 1	OQ189892	OQ164774	OQ164780	OQ164786	*Q. Fan 17910* (SYS), Renhua
*Commelinadanxiaensis* 2	OQ189893	OQ164775	OQ164781	OQ164787	*Q. Fan 18026* (SYS), Renhua
*Commelinadanxiaensis* 3	OQ189895	/	/	/	*Q. Fan 18232* (SYS), Renhua
*Commelinadiffusa*	OQ189891	OQ164776	OQ164782	OQ164788	*L.Y. Wang 1831* (SYS), Guangzhou
*Commelinaerecta*	MG639917.1	KJ772671.1	KR737134.1	KR735729.1	/
*Commelinapaludosa*	OQ189894	OQ164777	OQ164783	OQ164789	*Q. Fan 18247* (SYS), Renhua
*Commelinareptans*	KR733954.1	KR735150.1	KR736643.1	KR735386.1	/
*Commelinapurpurea*	/	GQ248103.1	EF590514.1	GQ248272.1	/
*Floscopascandens*	/	LC540553.1	AF312255.1	KY018930.1	/
*Murdanniaedulis*	/	MW617988.1	MW617988.1	MW617988.1	/
*Murdanniabracteata*	MT358291.1	/	/	/	/
